# The 2020 national diagnostic reference levels for nuclear medicine in Japan

**DOI:** 10.1007/s12149-020-01512-4

**Published:** 2020-08-27

**Authors:** Koichiro Abe, Makoto Hosono, Takayuki Igarashi, Takashi Iimori, Masanobu Ishiguro, Teruo Ito, Tomomasa Nagahata, Hiroyuki Tsushima, Hiroshi Watanabe

**Affiliations:** 1 Department of Radiology, Tokyo Medical University, 6-7-1 Nishishinjuku, Shinjuku-ku, Tokyo 160-0023 Japan; 2 Department of Radiology, Faculty of Medicine, Kindai University, 377-2 Ohno-Higashi, Osaka-Sayama, Osaka 589-8511 Japan; 3 Department of Radiological Technology, International University of Health and Welfare Narita Hospital, 852 Hatakeda, Narita-shi, Chiba 286-8520 Japan; 4 Department of Radiological Technology, Chiba University Hospital, 1-8-1 Inohana, Chuo-ku, Chiba-shi, Chiba 260-8677 Japan; 5 Department of Radiological Technology, Fujita Health University, 1-98 Dengakugakubo, Kutsukake-cho, Toyoake, Aichi 470-1192 Japan; 6 Department of Radiological Sciences, International University of Health and Welfare, 4-3 Kozunomori, Narita-shi, Chiba 286-8686 Japan; 7 Department of Radiological Technology, Osaka City University Hospital, 1-5-7, Asahi-machi, Abeno-ku, Osaka-shi, Osaka 545-8586 Japan; 8 Department of Radiological Sciences, Ibaraki Prefectural University of Health Sciences, 4669-2 Ami, Ami-machi, Inashiki-gun, Ibaraki 300-0394 Japan; 9 Department of Radiological Sciences, Faculty of Health Sciences, Gunma Paz University, 1-7-1 Tonyamachi, Takasaki-shi, Gunma 370-0006 Japan

**Keywords:** Diagnostic reference level, DRL, Nuclear medicine, Radiopharmaceutical, Hybrid CT

## Abstract

The diagnostic reference levels (DRLs) are one of several effective tools for optimizing nuclear medicine examinations and reducing patient exposure. With the advances in imaging technology and alterations of examination protocols, the DRLs must be reviewed periodically. The first DRLs in Japan were established in 2015, and since 5 years have passed, it is time to review and revise the DRLs. We conducted a survey to investigate the administered activities of radiopharmaceuticals and the radiation doses of computed tomography (CT) in hybrid CT accompanied by single photon emission computed tomography (SPECT)/CT and positron emission tomography (PET)/CT. We distributed a Web-based survey to 915 nuclear medicine facilities throughout Japan and survey responses were provided by 256 nuclear medicine facilities (response rate 28%). We asked for the facility's median actual administered activity and median radiation dose of hybrid CT when SPECT/CT or PET/CT was performed for patients with standard habitus in the standard protocol of the facility for each nuclear medicine examination. We determined the new DRLs based on the 75th percentile referring to the 2015 DRLs, drug package inserts, and updated guidelines. The 2020 DRLs are almost the same as the 2015 DRLs, but for the relatively long-lived radionuclides, the DRLs are set low due to the changes in the Japanese delivery system. There are no items set higher than the previous values. Although the DRLs determined this time are roughly equivalent to the DRLs used in the US, overall they tend to be higher than the European DRLs. The DRLs of the radiation dose of CT in hybrid CT vary widely depending on each imaging site and the purpose of the examination.

## Background

The use of radiation in the medical field continues to expand. Although patients who are exposed to radiation during their diagnoses and treatment often have the tremendous opportunity to overcome life-threatening illnesses, they are also at increased risk of radiation damage. Radiation-based diagnoses and treatment are justified only when the benefit is clearly greater than the risk associated with the radiation exposure (justification), and it is necessary to take measures to reduce radiation doses as much as possible during justified radiological diagnoses and treatment (optimization) [1]. The motivation to reduce and manage patients' radiation exposure as much as possible while maintaining the quality of radiation medicine is increasing worldwide [2].

In a survey conducted in the mid-1980s, British researchers reported that there were up to 50-fold differences in institutional radiation exposures to patients in domestic X-ray examinations [3], and they advocated the concept of guideline reference doses derived from patient-exposure doses in routine X-ray examinations. Each hospital was then able to compare its own doses with the guideline reference doses and identify the radiation exposure doses for which efforts could be most efficiently directed towards minimizing the patient dose. The concept of guideline reference doses was later established by the International Commission on Radiologic Protection (ICRP) [4–7] and the International Atomic Energy Agency (IAEA) [8]. With the progress of radiation generators and computer technology, a reference dose was set for each imaging modality, and these reference doses have become widely known as the diagnostic reference levels (DRLs) today.

The DRLs are determined by the following procedure: First, the most common protocol for each type of radiological examination performed at each institution is identified. Second, the median radiation exposure dose for ≥ 20 standard-size patients in the protocol is calculated. Third, the 75th percentile of the distribution of studied institutions for the median radiation exposure dose value is determined. Finally, the DRLs are established based on the 75th percentile [9]. In case the values thus derived were considered unreliable or inappropriate, the DRLs in the present study were established either by referring to the current DRLs, the drug package inserts or guidelines. Since the performance of radiological diagnostic equipment and the protocol of each radiological examination would vary depending on regional conditions and the era, DRLs should be established for each country or region, and should be reviewed periodically.

## Initiatives in Japan

The Japan Network for Research and Information on Medical Exposures (J-RIME) was established in 2010 to collect and share information on medical exposure studies and to discuss and implement measures for cooperation with international organizations. One of the first major projects of the J-RIME was the setting of DRLs, which provide a tool for the above-mentioned optimization. The J-RIME has collaborated with related societies and associations including the Japanese Society of Nuclear Medicine (JSNM) and the Japanese Society of Nuclear Medicine Technology (JSNMT). The J-RIME conducted a nationwide survey on the actual administered radioactivity in adults, and it established Japan's national DRLs in nuclear medicine in 2015 [10].

Because 5 years have already passed since the first DRLs were set, it is time to review the DRLs in light of the emergence of new drugs and the development of new technologies. Over the last 5 years, radiopharmaceuticals including ^111^In-pentetreotide were newly approved for use in Japan, and they were added as new items to the DRLs. Conversely, as the clinical uses of ^133^Xe gas, ^99m^Tc-HSA, and ^131^I-MIBG have been discontinued in Japan, these items were deleted from the DRLs. As the uses of single photon emission computed tomography (SPECT)/computed tomography (CT) and positron emission tomography (PET)/CT have become widespread, the radiation exposure doses provided by hybrid CT are also included in the 2020 DRLs.

## Nationwide survey targeting facilities, period, and method

The targets of the survey that we carried out were the hospitals, clinics, imaging centers, and other facilities that carry out nuclear medicine examinations across Japan. We created a survey form on the Web and asked each facility to enter its response to each item. The survey concerned the facility's nuclear medicine examinations conducted in July 2019, and each facility input on the Web the median of the actual administered activities of radiopharmaceuticals at their facility, plus the computed tomography dose index volume (CTDIvol) and dose length product (DLP) when hybrid CT was performed. The survey input period was from August 26, 2019 to September 24, 2019.

This work was exempted from an ethical review because (1) the recording and management of patient exposure are mandatory in nuclear medicine and some other radiological modalities in accord with provisions of the Ordinance for Enforcement of the Medical Care Act, and (*2*) this work collected and analyzed only median values of each institution from its existing data, without handling information of each patient or examination.

## How the new DRLs were determined

We asked 915 nuclear medicine facilities throughout Japan to complete a Web-based questionnaire, and 256 of the facilities entered their answers on the Web (response rate 28%).

We determined the new DRLs in nuclear medicine based on the following principles.

1. For radiopharmaceuticals, we asked each facility to enter the median actual administered activity of radioisotope (RI) for each nuclear medicine examination conducted for standard-size patients. This survey was conducted for adults only; for children aged ≤ 15 years, the consensus guidelines for pediatric nuclear medicine in Japan were developed by the JSNM in 2014 [11].
2. For the radiation doses of hybrid CT of SPECT/CT or PET/CT, we asked each facility to enter the median CTDIvol and DLP values for each nuclear medicine examination conducted for standard-size adults. For children aged ≤ 15 years, the CTDIvols and the DLPs were published as part of Japan's National 2020 DRLs [12].
3. The entered values were aggregated for type of nuclear medicine examination, and outliers that were apparently mistaken for input were excluded.
4. Regarding the administered activities of radiopharmaceuticals and the irradiation doses of hybrid CT, we calculated the 50th and 75th percentiles from the distributions of the values input by each facility.
5. For radiopharmaceuticals, for which values thus derived were considered unreliable or inappropriate, we determined the 2020 DRLs either by referring to the 2015 DRLs, drug package inserts, or current societal practice guidelines.
6. Finally, a reasonable value based on the 75th percentile was set as a two-digit integer value in principle.

## DRLs for administered activities of radiopharmaceuticals

The 2020 DRLs were determined as described above. For some items such as O-gas and amyloid PET, where the numbers of responses were < 10, we used the values shown in the 2015 DRLs or the drug package inserts as-is.

The survey results (50th and 75th percentiles), the determined 2020 DRLs, and the 2015 DRLs for each item are shown in Table 1 (radiopharmaceuticals for scintigraphy) and Table 2 (radiopharmaceuticals for PET). The radiopharmaceuticals or examination items that were newly added between the 2015 DRLs and the 2020 DRLs are ^99m^Tc-HSA-D for protein-losing gastroenteropathy, ^111^In-pentetreotide for somatostatin receptor imaging, ^18^F-FDG for aortitis, and amyloid tracers. The former three tracers were approved for Health Care Insurance coverage in Japan over the past 5 years. Because the three amyloid tracers were licensed for clinical use but not yet covered by insurance, examinations using these tracers were rarely performed and the number of survey responses was very small. There was no response from any facility about ^18^F-florbetaben. The DRLs for the amyloid tracers other than ^18^F-flutemetamol were thus determined based on the drug package inserts.

**Table 1:** Survey results and DRLs: radiopharmaceuticals for scintigraphy

Procedure and radiopharmaceutical	Median activity of radiopharmaceutical, MBq		2020 DRLs, MBq	2015 DRLs, MBq
	50th percentile	75th percentile		
Bone: ^99m^Tc-MDP	879.9	951.2	950	950
Bone: ^99m^Tc-HMDP	882.9	986.0	950	950
Bone marrow: ^111^In-chloride	76.0	76.9	80	120
Cerebral blood flow: ^99m^Tc-HM-PAO, rest or stress	760.0	871.1	800	800
Cerebral blood flow: ^99m^Tc-HM-PAO, rest and stress	1110.0	1116.0	1200	1200
Cerebral blood flow: ^99m^Tc-ECD, rest or stress	755.0	796.5	800	800
Cerebral blood flow: ^99m^Tc-ECD, rest and stress	1000.0	1088.0	1100	1100
Cerebral blood flow: ^123^I-IMP, rest or stress	183.0	206.1	200	200
Cerebral blood flow: ^123^I-IMP, rest and stress	249.8	272.0	270	300
Benzodiazepine receptor: ^123^I-iomazenil	185.0	195.5	200	200
Dopamine transporter: ^123^I-ioflupane	184.5	190.3	190	190
Cisternography: ^111^In-DTPA	38.0	38.2	40	70
Thyroid imaging: Na^123^I	8.1	8.7	10	10
Thyroid imaging: ^99m^Tc-O_4_^−^	185.0	238.5	240	300
Parathyroid: ^201^Tl- chloride	75.7	111.6	120	120
Parathyroid: ^99m^Tc-O_4_^−^	224.0	370.0	300	300
Parathyroid: ^99m^Tc-MIBI	745.5	828.1	800	800
Lung ventilation: ^81m^Kr-gas	185.0	190.6	200	200
Lung perfusion: ^99m^Tc-MAA	201.0	280.0	260	260
Venography: ^99m^Tc-MAA	370.0	477.5	500	500
Liver and spleen: ^99m^Tc-phytate	185.0	200.0	200	200
Liver function: ^99m^Tc-GSA	233.0	260.0	260	260
Hepatobiliary: ^99m^Tc-PMT	233.0	260.0	260	260
Liver and spleen: ^99m^Tc-Sn colloid	144.5	185.0	180	180
Myocardial perfusion: ^201^Tl- chloride	113.0	114.0	120	180
Myocardial perfusion: ^99m^Tc-tetrofosmin, rest or stress	740.0	836.0	840	900
Myocardial perfusion: ^99m^Tc-tetrofosmin, rest and stress	1077.2	1200.0	1200	1200
Myocardial perfusion: ^99m^Tc-MIBI, rest or stress	755.2	874.6	880	900
Myocardial perfusion: ^99m^Tc-MIBI, rest and stress	1076.4	1229.0	1200	1200
Myocardial fatty acid metabolism: ^123^I-BMIPP	124.0	129.1	130	130
Cardiac sympathetic nerve imaging: ^123^I-MIBG	126.0	129.9	130	130
Cardiac blood pool: ^99m^Tc-HSA-D	835.0	966.0	970	1000
Myocardial infarction: ^99m^Tc-PYP	740.0	809.5	800	800
Salivary gland: ^99m^Tc-O_4_^−^	209.4	370.0	370	370
Meckel's diverticulum: ^99m^Tc-O_4_^−^	370.0	432.8	440	500
Gastrointestinal bleeding: ^99m^Tc-HSA-D	933.0	1045.0	1040	1040
Protein losing gastroenteropathy: ^99m^Tc-HSA-D	932.0	1041.0	1040	–
Renal imaging, static: ^99m^Tc-DMSA	185.0	212.8	210	210
Renal imaging, dynamic: ^99m^Tc-MAG_3_	286.5	375.6	380	400
Renal imaging, dynamic: ^99m^Tc-DTPA	356.5	390.3	390	400
Adrenal cortex: ^131^I-Adosterol	37.0	39.4	40	44
Adrenal medulla: ^123^I-MIBG	128.1	132.4	130	130
Tumor: ^201^Tl-chloride	113.9	114.2	120	180
Tumor and inflammation: ^67^Ga-citrate	113.0	114.0	120	200
Somatostatin receptor: ^111^In-pentetreotide	156.0	185.7	120	–
Lymphatic system: ^99m^Tc-HSA-D	440.0	830.0	830	950
Sentinel lymph node, breast cancer: ^99m^Tc colloid	74.0	111.0	120	120
Sentinel lymph node, breast cancer: ^99m^Tc-phytate	72.8	100.0	120	120
Sentinel lymph node, melanoma: ^99m^Tc colloid	92.5	152.0	120	120
Sentinel lymph node, melanoma: ^99m^Tc-phytate	78.0	111.0	120	120
RI angiography: ^99m^Tc-HSA-D	932.0	1045.0	1000	1000

**Table 2:** Survey results and DRLs: radiopharmaceuticals for PET

Procedure and radiopharmaceutical	Median activity of radiopharmaceutical, MBq		2020 DRLs, MBq	2015 DRLs, MBq
	50th percentile	75th percentile		
Tumor: ^18^F-FDG, in-house-produced	220.2	235.1	240	240
Tumor: ^18^F-FDG, delivery	257.0	286.5	240	240
Tumor: ^18^F-FDG, per body weight	3.7	4.0	4	–
Aortitis: ^18^F-FDG, in-house-produced	238.0	254.5	240	–
Aortitis: ^18^F-FDG, delivery	249.5	276.9	240	–
Aortitis: ^18^F-FDG, per body weight	3.7	4.0	4	–
Myocardial metabolism: ^18^F-FDG, in-house-produced	226.0	259.0	240	240
Myocardial metabolism: ^18^F-FDG, delivery	259.0	322.1	240	240
Myocardial metabolism: ^18^F-FDG, per body weight	3.7	4.9	5	–
Myocardial perfusion: ^13^N-NH_3_	428.1	524.2	520	720
Brain: ^18^F-FDG, in-house-produced	196.0	223.2	240	240
Brain: ^18^F-FDG, delivery	230.0	254.9	240	240
Brain: ^18^F-FDG, per body weight	3.7	3.7	4	–
Amyloid: ^18^F-flutemetamol, in-house-produced	184.8	184.9	260^a^	–
Amyloid: ^18^F-flutemetamol, delivery	209.0	260.8	260	–
Amyloid: ^18^F-florbetapir, in-house-produced	370.0	370.0	370^b^	–
Amyloid: ^18^F- florbetapir, delivery	370.0	373.5	370^b^	–
Amyloid: ^18^F-florbetaben, in-house-produced	–	–	300^b^	–
^15^O–CO_2_ gas: 2D	270.0	270.0	8000^c^	8000
^15^O–O_2_ gas: 2D	390.0	390.0	6000^c^	6000
^15^O–CO gas: 2D	385.0	385.0	3000^c^	3000
^15^O–CO_2_ gas: 3D	1500.0	1800.0	1800^c^	2900
^15^O–O_2_ gas: 3D	2000.0	4500.0	4500^c^	7000
^15^O–CO gas: 3D	3000.0	3600.0	3600^c^	7500

^a^The DRL was determined to be same as in-house-produced ^18^F-flutemetamol

^b^DRLs were determined based on drug package inserts

^c^The 2015 DRLs were used as they were

Similar to the previous survey, we observed a tendency among the majority of nuclear medicine facilities; these facilities have been administering radiopharmaceutical activities based on the drug package inserts and guidelines, and many items in the 2020 DRLs are set equal to or lower than those in the 2015 DRLs. There are no items set higher than the previous values. Although this might be due to the advances in imaging devices and image reconstruction technology that have been obtained over the last five years, it is hoped that this is because the optimization of radiopharmaceutical use at each facility has been widely promoted by the establishment of the national DRLs.

Among the radiopharmaceuticals, the activity of ^111^In-chloride for bone marrow scintigraphy has largely decreased from 120 MBq in the 2015 DRLs to 80 MBq in the 2020 DRLs (a 33% decrease). Similarly, the activities of ^111^In-DTPA for cisternography, ^201^Tl scintigraphy for myocardium and tumor, and ^67^Ga scintigraphy for tumor or inflammation have decreased from 70 MBq in the 2015 DRLs to 40 MBq in the 2020 DRLs (a 43% decrease), from 180 to 120 MBq (a 33% decrease), and from 200 to 120 MBq (a 40% decrease), respectively. The main reasons for these decreases is likely to be the modifications of delivery systems made by manufacturers in 2016. Before then, Japanese manufacturers delivered radiopharmaceuticals with relatively long half-life nuclides such as ^111^In, ^201^Tl, and ^67^Ga a few days before the assay date. In many nuclear medicine facilities, the pharmaceuticals containing these RIs used to be administered without activity modification on the day they were delivered, resulting in administration activities that were higher than the assay activities. After 2016, as these pharmaceuticals are now delivered on the assay day, the latest DRLs are lower than the 2015 DRLs.

The DRLs that we determined herein are roughly equivalent to the US DRLs with the following exceptions: higher ^99m^Tc-MAA (260 vs. 215 MBq) and lower ^111^In-pentetreotide (120 vs. 237 MBq) and lower ^18^F-FDG (240 vs. 555 MBq for tumor, and 240 vs. 414 MBq for brain) [13]. The European DRLs are generally lower than those of the US and Japan [14]. Although differences in these DRLs can probably be attributed to differences in national/regional development status, customs, patient habitus and weight, etc., it might be necessary for each country/region to examine whether a given activity can be reduced with preserved image quality and whether nuclear medicine examinations have sufficient image quality with the currently used administration activities.

## DRLs for CT doses in hybrid CT

We also investigated hybrid CT examinations, i.e., SPECT/CT (Table 3) and PET/CT (Table 4) which were not examined in the previous survey, and we determined the DRLs of the CT irradiation dose. For the dose evaluation, we used the CTDIvol (mGy) and DLP (mGy cm) displayed on the CT console or recorded in the dose report. We prepared survey question for seven body regions (the whole body, brain, head and neck, chest, heart, abdomen and pelvis, and extremities) and for the examinations' purposes, e.g., attenuation correction (AC) only and AC + diagnosis (Dx) for SPECT/CT (Table 3). Compared to radiological diagnostic CT, SPECT/CT is still much less frequently performed at present, and CT is rarely performed for the purpose of AC alone even in brain and cardiac PET scans in Japan. In fact, there were only a few survey responses about brain and cardiac PET/CT in which CT was used for AC only. Regarding PET/CT, we asked CT dose for each three body region (whole body, brain, and heart), and the questions were divided into two purposes: clinical diagnosis (cl Dx) and cancer screening (Table 4).

**Table 3:** Survey results and DRLs: hybrid CT of SPECT/CT

Target site, purpose of use	CTDIvol, mGy			DLP, mGy cm		
	50th percentile	75th percentile	2020 DRLs	50th percentile	75th percentile	2020 DRLs
Whole body, AC + Dx	2.9	5.03	5.0	180	384.1	380
Brain, AC + Dx	10.6	22.60	23.0	262	419.2	410
Head and neck, AC + Dx	3.3	5.73	5.8	133	205.1	210
Chest, AC + Dx	2.6	4.06	4.1	90	164.8	170
Heart, AC + Dx	3.2	4.50	4.5	89	180.0	180
Abdomen, AC + Dx	3.0	5.02	5.0	89	206.8	210
Extremities, AC + Dx	3.2	4.69	4.6	159	237.2	230
Brain, AC only	7.3	12.86	13.0	140	350.9	330
Heart, AC only	1.6	4.04	4.1	45	84.1	85

**Table 4:** Survey results and DRLs: hybrid CT of PET/CT

Target site, purpose of use	CTDIvol, mGy			DLP, mGy cm		
	50th percentile	75th percentile	2020 DRLs	50th percentile	75th percentile	2020 DRLs
Whole body, cl Dx	4.2	6.02	6.1	451	608.6	600
Brain, cl Dx	19.6	30.75	31.0	384	641.3	640
Heart, cl Dx	5.5	9.03	9.1	209	388.9	380
Whole body, cancer screening	3.5	5.50	5.5	337	550.0	550

The path for establishing the DRLs of hybrid CT used in SPECT/CT and PET/CT is more complicated than that for establishing the DRLs of radiological diagnostic CT. In nuclear medicine, the aspects of an imaging protocol such as the imaged organ, imaged range, number of times of imaging, the purpose of the imaging, etc. differ depending on the type of examination, and the dose of CT exposure varies accordingly. In the UK, the Institute of Physics and Engineering in Medicine Working Party proposed national DRLs for hybrid CT that were determined for each examination type and purpose of CT [15]. Gardner et al. have developed four distinct dose mode ("low" for attenuation correction, "moderate" for localization, "standard" as diagnosis, and “metal” for orthopedic implants containing metal) based on scan purpose [16]. There is still room for further considerations of how to classify the DRLs for hybrid CT.

A systematic review published in 2018 introduced 14 articles for determining the DRLs for PET/CT and SPECT/CT [17]. Among 14 PET/CT and five SPECT/CT articles, five PET/CT and two SPECT/CT studies determined the DRLs for hybrid CTs. The values of CTDIvol and DLP varied widely as follows. For PET/CT: CTDIvol 4.3–10.2 mGy and DLP 400–750 mGy cm; for SPECT/CT: CTDIvol 2.1–5.9 mGy of CTDIvols and DLP 36–240 mGy cm. Compared to these values, the CTDIvol and DLP for PET/CT obtained in the present survey are similar at 5.5–31.0 mGy and 380–640 mGy cm, respectively. However, these values for SPECT/CT in the present survey tend to be high at CTDIvol 4.1–23.0 mGy and DLP 85–380 mGy cm. As mentioned above, it seems that these differences are due largely to the differences in CT devices and protocols. We look forward to an opportunity to further reduce patient radiation exposure by comparing other DRLs with a detailed consideration of imaging protocols.

## Limitations

One of the limitations of this study is that there were items for which very few survey responses were obtained. The ICRP recommends using the data for ≥ 10–20 facilities for the establishment of local DRLs [5]. Because responses from only a few nuclear medicine facilities were available, we determined the DRLs for amyloid and O-gas PET based on the drug package inserts and the 2015 DRLs. Moreover, the survey excluded items with ≤ 10 responses, resulting in the removal of most of the special-purpose items of hybrid CT. Another limitation is that the survey did not take into account the manufacturing date of the imaging device or the accompanying new technologies. Current advanced techniques, such as semiconductor cameras, time-of-flight, and point spread function technologies, etc. used in SPECT and/or PET, plus the automatic tube current modulation technique, iterative image reconstruction algorithms, and more used in CT can significantly reduce patients' radiation exposure without deterioration of the image quality. Whether each facility adopts these technologies would have a significant impact on the patient exposures.

## Future outlook for DRLs

Although DRLs are becoming an indispensable index for optimizing examination protocols and reducing patient radiation exposure, problems concerning their uses and limitations have been pointed out. The achievable dose (AD) corresponding to the 50th percentile of the used dose has been recommended for use as an effective tool for advancing optimization while ensuring image quality [18]. The administered activity duration product (ADP), which is the product of the administration activity (MBq) and the acquisition time (min), has been proposed as a better measure for optimization with preservation of the image quality [17, 18]. Rehani proposed a new indicator called the acceptable quality dose (AQD), which takes both image quality and patient size into consideration [19]]. We would like to further promote the use of DRLs while utilizing and incorporating these concepts, all of which are designed to reduce or limit patients' radiation exposure.
